# Nonfermenting, Gram-Negative Bacilli Causing Neonatal Sepsis in Odisha, India: Four-Year Surveillance

**DOI:** 10.7759/cureus.22219

**Published:** 2022-02-14

**Authors:** Santosh K Panda, Manas K Nayak, Pravati Jena, Soumini Rath, Ramakrushna Gudu, Rishabh Pugulia, Subhra Snigdha Panda

**Affiliations:** 1 Neonatology, Kalinga Institute of Medical Sciences, Bhubaneswar, IND; 2 Pediatric Medicine, Kalinga Institute of Medical Sciences, Bhubaneswar, IND; 3 Pediatrics, Kalinga Institute of Medical Sciences, Bhubaneswar, IND; 4 Microbiology, Kalinga Institute of Medical Sciences, Bhubaneswar, IND

**Keywords:** preterm, mortality, multi drug resistant, sepsis, non formenters, neonates

## Abstract

Introduction

In India, blood culture-positive sepsis results in mortality in 33%-35% of affected neonates. Nonfermenting Gram-negative bacilli (NFGNB), particularly *Acinetobacter* *baumannii *and *Burkholderia cepacia* commonly cause hospital-acquired infection.

Materials and methods

We performed a subgroup analysis as part of a prospective study conducted in a neonatal intensive care unit in a tertiary care hospital in Odisha, India, between January 2017 and December 2020. Neonates with blood culture-positive sepsis caused by NFGNB were enrolled in this study. Demographic characteristics of the neonates, clinical features of sepsis, complications, need for supportive care, and blood culture sensitivity patterns were recorded and analyzed.

Results

A total of 168 organisms were isolated in blood cultures during our study period, of which 48 (29%) were NFGNB species. Among these 48 species, *A. baumannii *(37.5%) and *B.* *cepacia* (33.3%) were the most common NFGNB in our study. Neonates with sepsis commonly exhibited feeding intolerance (64.5%), circulatory insufficiency that necessitated vasopressor treatment (54.1%), disseminated intravascular coagulopathy (35.4%), seizures (33.3%), and the need for respiratory support (56.2%). NFGNB were multidrug-resistant (MDR) in 70.8% of cases, and 93.7% of *B. cepacia* and 55.5% of *A. baumannii *were MDR.

Conclusions

*A.* *baumannii *and *B. cepacia* are NFGNB commonly isolated in neonatal cases of blood culture-positive sepsis. The prevalence of MDR NFGNB sepsis is gradually increasing, which poses a threat to neonates. Strict aseptic precautions and antibiotic stewardship are thus mandatory in perinatal practice.

## Introduction

Sepsis accounts for approximately one-third of neonatal deaths in India [1,2]. Blood culture-positive sepsis results in mortality in 33%-35% of affected neonates [3]. The burden of clinical sepsis is higher in India (17,000/100,000 live births) in comparison with annual global data (2,202/100,000 live births) [4,5]. *Klebsiella* and *Escherichia coli* are the bacteria most commonly responsible for sepsis in India and South Asia, and nonfermenting Gram-negative bacilli (NFGNB), particularly *Acinetobacter baumannii* and *Burkholderia cepacia* commonly cause hospital-acquired infection [6,7]. Literature about clinical outcomes of infections with individual NFGNB is available, but publications about the profile of all NFGNB that cause sepsis and its outcome are limited. An increasing incidence of multidrug-resistant (MDR) NFGNB sepsis in our NICU prompted us to study the clinical outcomes of NFGNB culture-positive sepsis and antibiotic resistance among NFGNB in a tertiary care institution.

## Materials and methods

This prospective study was conducted in a neonatal intensive care unit in a tertiary care hospital in Odisha, India, between January 2017 and December 2020. Subgroup analysis of the study was done. Neonates (<28 days after birth, and <44 weeks of corrected gestational age if born prematurely) with blood culture-positive sepsis caused by NFGNB were enrolled in this study after approval by our institute's ethics committee.

Blood cultures were obtained whenever a neonate exhibited signs and symptoms of sepsis, such as lethargy, refusal to feed, apnea, seizures, temperature instability, respiratory distress, and poor perfusion with shock, or when asymptomatic neonates were admitted with risk factors for sepsis (maternal fever or chorioamnionitis; prolonged rupture of the membranes for >24 h in preterm infants <35 weeks of gestational age; and foul-smelling amniotic fluid [8]. Approximately 1-2 mL of peripheral venous blood was collected in a BD BACTEC Peds Plus blood culture vial via aseptic technique, and the blood was cultured in a BD BACTEC FX culture system (Becton Dickinson, Sparks, MD, USA). Blood culture-positive neonatal sepsis was diagnosed when a microorganism was isolated in a sample from a neonate who had clinical features or maternal risk factors suggestive of sepsis [7,9]. We used the Kirby-Bauer disk diffusion method on Mueller-Hinton agar for antibiotic susceptibility testing, and we interpreted the results in accordance with guidelines of the Clinical & Laboratory Standards Institute [10]. Dehydrated media and antibiotic disks (HiMedia Laboratories Pvt Ltd, Mumbai, India) were used for culturing. The minimum inhibitory concentration value was determined through an automated disk diffusion method (VITEK 2 Compact; bioMérieux, Marcy-l'Étoile, France) for different groups of antibiotics. The sensitivity or resistance pattern of the isolated organisms was reported. The Gram-negative bacteria were tested for susceptibility to various antibiotics: piperacillin-tazobactam, aminoglycosides (gentamicin or amikacin), extended-spectrum cephalosporins (ceftazidime, cefoperazone plus sulbactam, or cefotaxime), fluoroquinolones (ciprofloxacin), carbapenems (imipenem or meropenem), and colistin. Then, these bacteria were classified according to their resistance pattern [11].

The demographic characteristics of neonates, perinatal risk factors, intramural and extramural delivery, mode of delivery, signs, symptoms, and need for supportive care management such as respiratory support, vasoactive drugs, and blood component transfusion were recorded in a pre-designed proforma document. The blood culture data, such as the type of microorganisms (Gram-negative bacteria, Gram-positive bacteria, or fungi) and their resistance patterns, were documented. On the basis of time onset of signs and symptoms (before or after 72 h of postnatal life), sepsis was classified as early-onset or late-onset neonatal sepsis, respectively [6,7].

Associated organ-specific morbidities such as pneumonia, meningitis, acute kidney injury, thrombocytopenia, disseminated intravascular coagulation (DIC), shock, necrotizing enterocolitis, and multiple-organ dysfunction syndrome were recorded. The shock was diagnosed when circulatory insufficiency that necessitated fluid bolus or supportive vasoactive drugs was present [12]. Acute kidney injury was diagnosed according to the neonatal guidelines of Kidney Disease Improving Global Outcomes [13]. DIC was diagnosed when both thrombocytopenia (platelet count of <150,000/mm^3^) and coagulopathy (international normalized ratio of >1.5 or activated partial thromboplastin time of >49 s) were present [14]. Multiple-organ dysfunction syndrome, necrotizing enterocolitis, and healthcare-associated infection were diagnosed according to standard guidelines [15-17]. The final outcome, such as recovery, death, or leaving against medical advice, was recorded. Mortality was defined as infection-attributable death that occurred before clinical features of bacteremia were resolved in a neonate with culture-positive sepsis [18].

Microsoft Excel 2013 (Microsoft, Redmond, WA, USA) was used for data collection, and descriptive statistics such as percentages, means ± standard deviations, and medians (with interquartile ranges) were used to describe variables. We used SPSS Statistics for Windows, version 21.0 (IBM Corporation, Armonk, NY, USA) to perform statistical analyses.

## Results

A total of 168 blood culture-positive organisms were isolated during our study period, of which 48 (29%) were NFGNB species. *A. baumannii* (37.5%) and *B. cepacia* (33.3%) were the most common NFGNB in our study, followed by *Pseudomonas aeruginosa *(10.4%), *Serratia marcescens* (8.3%), *Elizabethkingia meningoseptica* (4.1%), *Sphingomonas paucimobilis* (4.1%), and *Salmonella typhi* (2%).

The mean birth weight of neonates with sepsis was 1.84 ± 0.87 kg, and the mean gestational age was 33.47 ± 4.79 weeks. Tables 1, 2 list the demographic characteristics of the infants and identifiable risk factors for NFGNB sepsis, respectively.

**Table 1:** Demographic characteristics of 48 infants with NFGNB sepsis NFGNB, nonfermenting Gram-negative bacilli; VLBW, very low birth weight.

Variable	Total no of cases
Female-to-male ratio	15:33
No. of infants with VLBW (<1.5 kg)	23 (47.9%)
No. of infants with LBW (<2.5 kg)	36 (75%)
No. of preterm infants (<37 weeks of gestational age)	29 (60.4%)
No. of infants with perinatal asphyxia (Apgar scores of <7 at 5 min)	12 (25%)
No. of infants born by cesarean delivery	18 (37.5%)
No. of extramural deliveries	34 (70.8)
No. of infants with early-onset sepsis	34 (70.8%)
No. of infants with late-onset sepsis	14 (29.2%)

**Table 2:** Identifiable risk factors in NFGNB sepsis NFGNB, nonfermenting Gram-negative bacilli.

Risk factors	No. of neonates (n= 48)
Premature rupture of membranes	15 (31.2%)
Chorioamnionitis	17 (35.4%)
Prematurity	29 (60.4%)
Need for central catheters	27 (56.2%)
Need for mechanical ventilation	20 (41.6%)
Patent ductus arteriosus	9 (18.7%)

Neonates with sepsis commonly exhibited feeding intolerance (64.5%), circulatory insufficiency that necessitated vasopressor treatment (54.1%), DIC (35.4%), seizures (33.3%), and a need for respiratory support (56.2%; Table 3).

**Table 3:** Complications and survival outcomes of NFGNB infections NFGNB, nonfermenting Gram-negative bacilli.

Neonatal morbidity	No. of neonates (n=48)
Feeding intolerance	31 (64.5%)
Temperature instability	7 (14.5%)
Pneumonia	7 (14.5%)
Need for platelet transfusion	12 (25%)
Shock	26 (54.1%)
Disseminated intravascular coagulopathy	17 (35.4%)
Meningitis	13 (27%)
Seizure	16 (33.3%)
Acute kidney injury	7 (14.5%)
Necrotizing enterocolitis	7 (14.5%)
Respiratory support	27 (56.2%)
• Noninvasive	7 (14.5%)
• Invasive	20 (41.7%)
Mortality	2 (4.1%)

The antibiotics to which NFGNB were resistant are listed in Table 4. NFGNB were MDR in 70.8% of cases; 93.7% of *B. cepacia* and 55.5% of *A. baumannii* were MDR.

**Table 4:** Antimicrobial-resistant pattern of nonfermenting Gram-negative bacilli

Antibiotic	No. of infants with drug-resistant infection (n= 48)	Percentage (%)
Aminoglycosides	24	50
Cephalosporins	16	33.3
Piperacillin–tazobactam	32	66.6
Meropenem	16	33.3
Fluoroquinolones	18	37.5
Colistin	22	45.8
Multiple drugs	34	70.8

## Discussion

We found that in nearly one-third of cases, neonatal sepsis was caused by NFGNB in our neonatal intensive care unit in a tertiary care hospital. *A. baumannii* and *B. cepacia* were the NFGNB that caused most cases of both early- and late-onset neonatal sepsis. Survival outcomes were better in our study than in previously reported studies.

Low birth weight and preterm birth were the most common demographic characteristics among neonates with NFGNB sepsis as in those other studies [19,20]. These characteristics may explain the prolonged requirement for total parenteral nutrition and supportive devices such as mechanical ventilators and central catheters, which are sources of hospital-acquired infections. The involvement of *A. baumannii* and *B. cepacia* in early-onset neonatal sepsis is a major concern; such infections can be acquired either from maternal flora during the intrapartum period or from elsewhere during the immediate postnatal period. In our study, NFGNB sepsis was more common among hospital-born neonates than among those born at home like that in the study by Viswanathan et al. [21]. Because NFGNB can survive in a hospital environment, multiple strategies to prevent sepsis are needed.

Approximately two-thirds of the NFGNB that we found were MDR, and the failure of commonly used antibiotics in sepsis management is alarming. In this study, NFGNB were resistant to amikacin (in 50% of cases), piperacillin-tazobactam (in 66.6%), carbapenems (in 33.3%), and colistin (in 45.8%). Recently, a high prevalence of MDR Gram-negative bacteria was found in two studies in North India [7,12]. The incidence of multiple-drug resistance in our study was highest among *S. marcescens* (100%), followed by *B. cepacia* (93.7%), *A. baumannii* (55.5%), and *P. aeruginosa* (40%); these findings are similar to those of Viswanathan et al. [21]. The prevalence of sepsis caused by MDR Acinetobacter spp. is higher among neonates delivered outside hospitals (91%) than among those delivered in hospitals (82%) [6,7].

We found colistin resistance in 81.2% of *B. cepacia *and 5.5% of *A. baumannii*, whereas other studies have shown that 98%-100% of Gram-negative organisms are sensitive to colistin [7,22]. In contrast, the incidence of carbapenem resistance (38% of *A. baumannii *and 25% of *B. cepacia*) was lower in our study than in other studies [23]. The rise in antimicrobial resistance among Gram-negative organisms that cause neonatal sepsis may be attributable to the increased use of colistin in the past decade and will pose a formidable threat to newborns in the future [24,25].

The survival rate in our study was 95.9%, whereas mortality rates were higher (23%-50%) in previous studies of blood culture-proven sepsis [3,6,7,18]. The fatality rates among neonates with isolated *A. baumannii* and *B. cepacia* infections were as high as 38%-59% and 6.3%-17%, respectively [6,7,26,27]. The survival rates in our study may have been better because of early detection of the clinical features of intramural early-onset sepsis, aggressive supportive care, and the use of empirical antibiotics recommended in previous antibiograms at our hospital.

## Conclusions

*A. baumannii* and *B. cepacia* are NFGNB commonly isolated in blood cultures from neonates with sepsis, and preterm infants are more susceptible to such infections than full-term infants. The incidence of sepsis caused by MDR strains of NFGNB is rising among newborns, which may result from the increased use of antibiotics such as carbapenems and colistin, and it will pose a formidable threat to newborns in the future. Timely aseptic precautions and strict antibiotic stewardship are thus mandatory in perinatal practice.
